# Supplementary figures and images for: Projections of adult skills and the effect of COVID-19
Source: PLoS One. 2022 Nov 23;17(11):e0277113. doi: 10.1371/journal.pone.0277113 (PMC9683630; doi:10.1371/journal.pone.0277113)

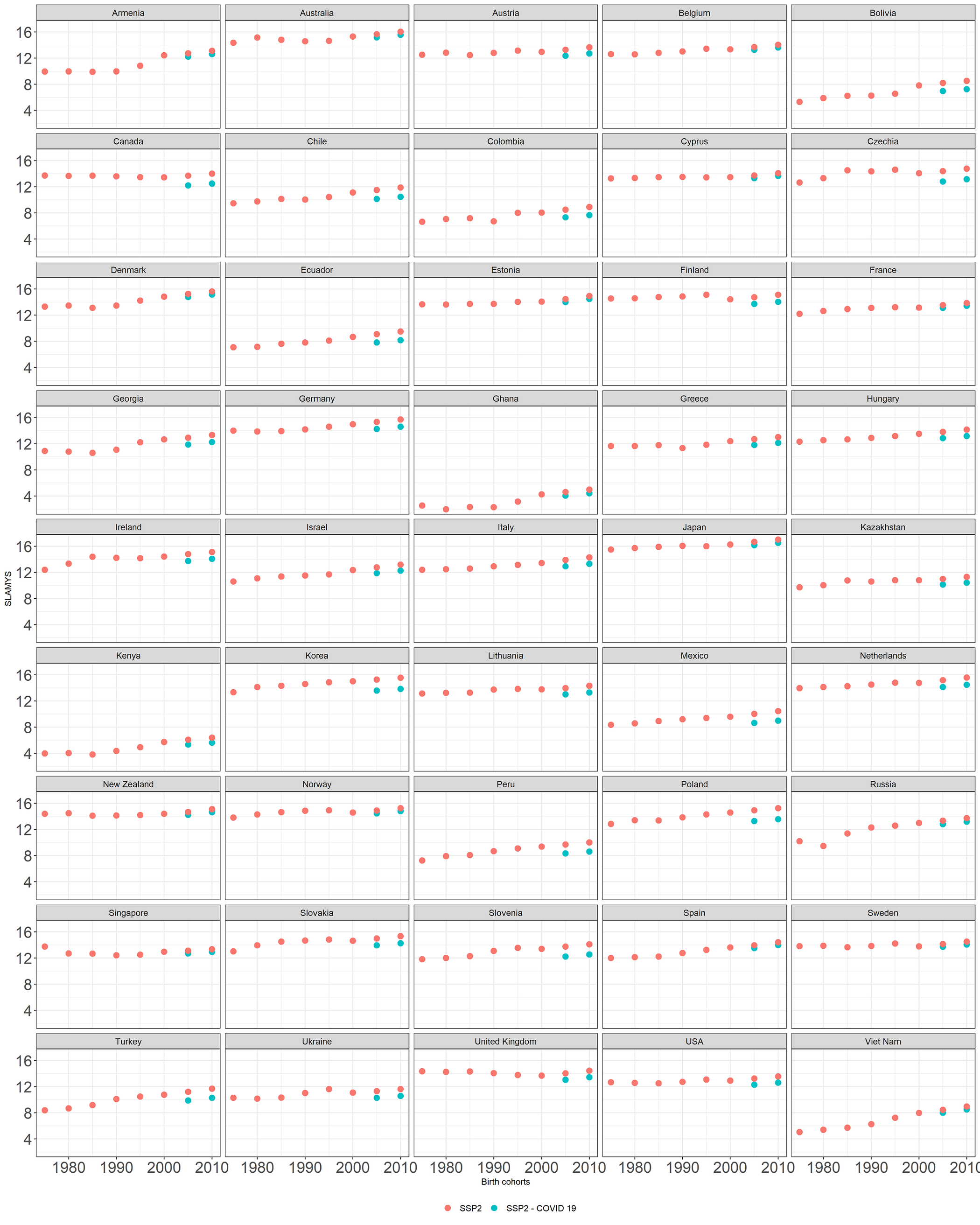

Supplement: S1 Fig — Source: Authors’ own calculations. (TIF) [file pone.0277113.s001.tif]
