# Supplementary material for: Projections of adult skills and the effect of COVID-19
Source: PLoS One. 2022 Nov 23;17(11):e0277113. doi: 10.1371/journal.pone.0277113 (PMC9683630; doi:10.1371/journal.pone.0277113)
Supplement: Attachment — Submitted filename: response_to_reviewers.docx [file pone.0277113.s002.docx]

Dear Editor and Reviewers,

We are pleased to submit the revised version of our original research article entitled “Projections of adult skills and the effect of COVID-19” to be considered for publication in PlosOne.

In this submission, we address the suggestions from the reviewers. First of all, we would like to thank for the constructive comments and suggestions. As per the editorial comment about Figure 2: The map used in the figure was constructed by the authors using R software. The base map is constructed using Natural Earth public domain maps, we added information about the R packages, cited Natural Earth web site and data used in drawing the map in the figure notes.

To meet the points raised by Reviewer #1, the Discussion & Conclusion section has been extended. A discussion about the increasing global skills gap is added in the second paragraph and relevant references were cited. Another discussion about previous evidence of learning loss was added in the third paragraph with the relevant citations.

Reviewer #2 pointed out the uncertainty about the number of projection scenarios used. It had been mentioned in the first paragraph of the Results section. Now, it is clearly stated once again in the first paragraph of the Discussion & Conclusion section.

In answer to the point of Reviewer #3 about the link to health, we have added a line in the Discussion & Conclusion section, reflecting on the potential long-term consequences of the pandemic on mental health. A detailed discussion would go, however, beyond the scope of this paper.

Thank you for your consideration,

The Authors
